# Pulmonary artery acceleration time accuracy for systolic pulmonary artery pressure estimation in critically ill patients

**DOI:** 10.1186/s13089-022-00276-4

**Published:** 2022-06-20

**Authors:** Valentino Dammassa, Francesco Corradi, Costanza Natalia Julia Colombo, Francesco Mojoli, Susanna Price, Guido Tavazzi

**Affiliations:** 1 University of Pavia, Pavia, Italy; 2 Adult Intensive Care Unit, Royal Brompton Hospital, London, UK; 3 Department of Surgical, Medical and Molecular Pathology and Critical Care Medicine, University of Pisa, Pisa, Italy; 4 Anaesthesia and Critical Care Medicine. E.O. Ospedali Galliera, Genoa, Italy; 5 Anaesthesia and Intensive Care, Fondazione IRCCS Policlinico San Matteo, Pavia, Italy; 6 Department of Clinical-Surgical, Diagnostic and Paediatric Sciences, Unit of Anaesthesia and Intensive Care, Fondazione IRCCS Policlinico San Matteo, University of Pavia, Pavia, Italy

**Keywords:** Pulmonary artery acceleration time, Pulmonary artery pressure, Acute cardiovascular failure, Acute respiratory failure, Right ventricular dysfunction

## Abstract

**Background:**

Estimation of pulmonary pressures is of key importance in acute cardiovascular and respiratory failure. Pulmonary artery acceleration time (PAAT) has emerged as reliable parameter for the estimation of systolic pulmonary artery pressure (sPAP) in cardiological population with preserved right ventricular function. We sought to find whether PAAT correlates with sPAP in critically ill patients with and without right ventricular (RV) systolic dysfunction.

**Methods:**

*Observational study*. We measured sPAP using continuous-wave Doppler analysis of tricuspid regurgitation velocity peak method and we assessed the validity of PAAT in estimating sPAP in patients admitted to adult intensive care unit (ICU) for acute cardiovascular and respiratory failure.

**Results:**

We enrolled 236 patients admitted to cardiothoracic ICU for cardiovascular and respiratory failure (respectively: 129, 54.7% and 107, 45.3%). 114 (48.3%) had preserved RV systolic function (defined as TAPSE ≥ 17 mm), whilst 122 (51.7%) had RV systolic impairment (defined as TAPSE < 17 mm). A weak inverse correlation between PAAT and sPAP (ρ–0.189, *p* 0.0035) was observed in overall population, which was confirmed in those with preserved RV systolic PAAT and sPAP (ρ–0.361, *p* 0.0001). In patients with impaired RV systolic function no statistically significant correlation between PAAT and sPAP was demonstrated (*p* 0.2737). Adjusting PAAT values for log_10_, heart rate and RV ejection time did not modify the abovementioned correlations.

**Conclusions:**

PAAT measurement to derive sPAP is not reliable in cardiothoracic critically ill patients, particularly in the coexistence of RV systolic impairment.

## Background

Pulmonary hypertension (PH) is a condition defined by an increase in afterload leading to a mean pulmonary artery pressure (mPAP) ≥ 25 mmHg at rest [1]. The presence of right ventricular (RV) dysfunction is associated with significant increase in mortality [1, 2]. Although, the diagnosis of PH requires right-heart catheterization, echocardiography is recommended as a first-line non-invasive diagnostic investigation for PH assessment (Class I, C) [1].

A number of echocardiographic parameters have been validated to assess RV morphology and function and to estimate pulmonary pressures [3]. Continuous-wave Doppler analysis of tricuspid regurgitation velocity peak (TRV_max_) is considered the cornerstone of non-invasive assessment of systolic pulmonary artery pressure (sPAP) [1]. Although this method has shown a good correlation with invasive measurements, it is burdened with some limitations, including the dependency on a well-defined envelope of tricuspid regurgitation (TR) and an adequate alignment with Doppler signal [4, 5].

Pulmonary artery acceleration time (PAAT) has demonstrated good reliability in estimating mPAP [6, 7] and, recently, it has been considered an appealing alternative to TR-based method in the estimation of sPAP [8]. However, there is lack of data regarding the accuracy of this parameter in patients with RV dysfunction in acute setting.

We sought to investigate whether PAAT estimation of sPAP is reliable also in critically ill patients admitted to intensive care unit (ICU) for acute cardiovascular failure and respiratory failure related to moderate-to-severe acute respiratory distress syndrome (ARDS), with and without RV systolic dysfunction, defined as tricuspid annular plane systolic excursion (TAPSE) < 17 mm [9].

## Methods

The project has been approved by the ethical committee of Royal Brompton Hospital NHS Foundation Trust (London, UK), where it was carried out. Written informed consent was acquired for all patients. The study was conducted on consecutive patients admitted to adult cardiothoracic ICU for post-cardiac surgery and respiratory failure related to at least moderate ARDS (PaO_2_ on FiO_2_ ratio below 200).

We measured sPAP using the TRV_max_-method [1] and we assessed the validity of PAAT in estimating sPAP as previously described [8].

Exclusion criteria were: TR envelope judged as insufficient to assess the sPAP, pulmonary prothesis, tricuspid surgery, congenital heart disease and significant pulmonic valvular stenosis (as defined by a continuous-wave peak jet velocity ≥ 2 m/s across the pulmonic valve). Images were recorded in multiple views thus obtaining optimal imaging for the primary analysis.

TAPSE and tissue Doppler S’ was measured in the apical four-chamber view during systole with cursor placed through the lateral tricuspid annulus. In accordance with current guidelines, TAPSE < 17 mm and S’ < 9.5 cm/sec were regarded as pathological [10].

Continuous-wave Doppler was used to measure the peak velocity of the TRV_max_. sPAP was determined from the TR jet velocity using the simplified Bernoulli equation adding the right atrial pressure (RAP), directly measured from central venous pressure (at end-expiration) or estimated from inferior vena cava (IVC) diameter and respiratory collapsibility [11, 12].

**Fig. 1:**
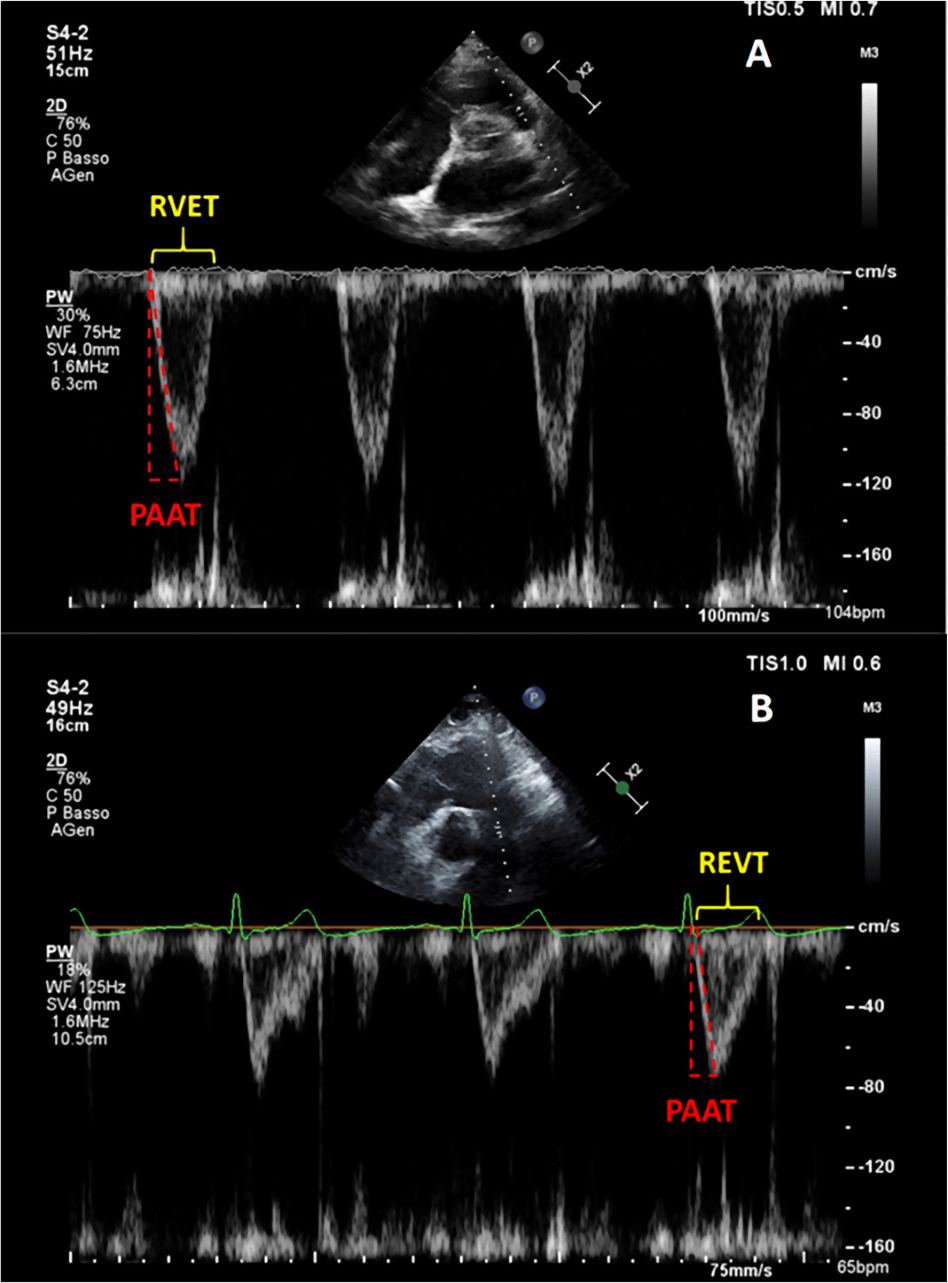
Pulmonary artery acceleration time (PAAT, dashed red line) and right ventricular ejection time (RVET, yellow brace) measurement in parasternal short-axis view (**A**) and subcostal view (**B**). PAAT is calculated as the time interval between the onset of systolic pulmonary artery flow and its peak flow velocity; RVET is measured from the onset to the end of pulmonary artery Doppler spectrum. For further adjustment, sPAP was derived on the basis of the linear correlation linking PAAT to TRV_max_ as follows: log_10_(sPAP) = −0.004 (PAAT) + 2.1 [8]. Mean PAP (mPAP) can be estimated using the following formulae proposed by Dabestani et al. [28]: if PAAT ≥ 120 ms, mPAP = 79 – (0.45 * PAAT); if PAAT < 90 ms, mPAP = 90 – (0.62 * PAAT)

Doppler interrogation of the proximal pulmonary artery was performed either in parasternal long-axis outflow and/or the parasternal short-axis (Fig. 1A) in order to exclude pulmonic stenosis and to assess pulmonary artery flow; for patients with poor acoustic parasternal window short-axis subcostal view was preferred (Fig. 1B). Pulmonary artery flow was evaluated by placing the pulsed-wave Doppler sample volume at the pulmonary valve annulus: PAAT was calculated as the interval between the onset of ejection and the peak flow velocity. PAAT have also been indexed by RV ejection time (RVET) [13] and heart rate (HR), dividing its value for square root of RR interval [14, 15].

All values used for analysis represented the average of three consecutive cardiac cycles, with the exception of patients with atrial fibrillation, in whom five-beat averages were obtained. Continuous-wave Doppler was used with a sweep speed of 100 mm/s to achieve a satisfactory envelope.

All measurements were made offline. Echocardiograms were interpreted by fully certified physicians who were blinded to the patients’ medical history and diagnosis.

### Statistical analysis

Results of continuous data were expressed as mean ± standard deviation for normally distributed data and median (interquartile range) for values not normally distributed; normal distribution of data was assessed with D’Agostino–Pearson test. Categorical variables were expressed as percentage.

Categorical data were compared by Pearson’s *χ*2 test with Yates correction or Fisher’s exact test when appropriate. Continuous variables were compared with Mann–Whitney *U*-test for unpaired data. Relationships between PAAT and haemodynamic indexes were evaluated by Spearman correlation coefficient. A two-sided *p*-value < 0.05 was assumed as statistically significant.

Statistical analyses were performed by using SPSS software (version 20.0; SPSS Inc, Chicago, IL). The intra-observer and inter-observer variability of PAAT and TRV_max_ were expressed as intra-class correlation coefficient (ICC), which was derived using a one‐way random‐effects model.

## Results

### Patients characteristics

We consecutively enrolled 255 adult patients admitted to cardiothoracic ICU. Nineteen subjects were excluded for inadequate echocardiographic windows, thus 236 patients were considered for the final analyses. The mean age was 63 ± 17 years, and 150 (63.5%) were males. 114 (48.3%) had preserved RV systolic function (defined as TAPSE ≥ 17 mm), whilst 122 (51.7%) had RV systolic impairment (defined as TAPSE < 17 mm). One hundred and twenty-nine patients (54.7%) were admitted after cardiac surgery, while 107 (45.3%) for respiratory failure related to moderate-to-severe ARDS. Amongst those undertaking cardiac surgery: 78 (60.5%) underwent mitral valve replacement, 32 (24.8%) aortic valve replacement and 19 (14.7%) coronary artery bypass graft. Two hundred and twenty patients had RAP derived from invasive measurements.

Stratifying for the cause of ICU admission, a statistically significant difference in the incidence of RV dysfunction was observed: 69.8% of patients affected by acute cardiovascular failure experienced RV dysfunction, while only 29.9% had RV dysfunction in the respiratory failure subgroup (*p* < 0.001). Need for positive-pressure ventilation (both invasive and non-invasive) and respiratory gas exchanges (PaO_2_ on FiO_2_ ratio and PaCO_2_) did not differ between the two cohorts. No patients required mechanical circulatory support nor inhaled nitric oxide at time of echocardiographic assessment.

Echocardiographic parameters tested and their derived variables, except for RAP and HR-corrected PAAT, shown a statistically significant difference between preserved and impaired RV function (Table 1). As shown in Table 2, patients admitted to ICU for acute cardio-circulatory failure were characterized by lower TAPSE and sPAP and shorter PAAT.

**Table 1:** Demographic, clinical characteristics and echocardiographic parameters of overall population and the two cohorts

	Overall population	TAPSE ≥ 17 mm	TAPSE < 17 mm	*p*-value
Population characteristics				
Number of patients	236 (100%)	114 (48.3%)	122 (51.7%)	–
Age (years)	63 ± 17	62 ± 18	64 ± 16	0.3672
Males, n (%)	150 (63.5%)	69 (60.5%)	81 (66.4%)	0.3477
BSA (m^2^)	1.86 ± 0.22	1.90 ± 0.23	1.83 ± 0.20	0.0131
APACHE II	12.9 ± 5.0	12.7 ± 4.5	13.1 ± 5.4	0.5386
Cause of ICU admission				
Respiratory failure, n	107 (45.3%)	75 (70.1%)	32 (29.9%)	–
Post-cardiac surgery, n	129 (54.7%)	39 (30.2%)	90 (69.8%)	–
Ventilation and gas exchanges				
Mechanical ventilation (included NIV), n	198 (83.9%)	98 (86.0%)	101 (82.8%)	0.4999
PEEP (cmH_2_O)	9.0 (7.0–11.0)	10 (7.8–11.0)	8.0 (6.0–10.0)	0.0796
PaO_2_/FiO_2_ (mmHg)	191.1 (139–221.5)	187 (126.5–216.0)	198 (155.8–228.3)	0.1012
PaCO_2_ (mmHg)	43.0 (38.0–49.0)	44.0 (40.0–51.5)	42 (37.0–49.0)	0.0955
Echocardiographic parameters and derived variables				
TAPSE (mm)	16.0 (10.0–21.0)	21.0 (19.0–24.0)	10.1 (8.0–14.0)	< 0.0001
S’ (cm/Sec)	9.4 (6.5—12)	11.5 (9.4–15.2)	6.9 (4.2–9.2)	< 0.0001
RAP (mmHg)	8.0 (8.0–10.0)	8.0 (8.0–10.0)	10.0 (8.0–10.0)	0.2338
sPAP (mmHg)	48.1 (39.9–58.7)	51.7 (41.2–61.7)	44.8 (39.0–52.9)	0.0020
PAAT (ms)	87.5 (74.0–98.0)	94.0 (77.0–106.0)	85.0 (74.0–92.0)	0.0009
Log_10_PAAT	1.94 (1.87–1.99)	1.97 (1.89–2.03)	1.93 (1.87–1.96)	0.0008
HR (bpm)	84 (72 -97)	80 (67–90)	90 (76–100)	0.0002
RVET (ms)	229.0 (193.0–265.5)	222.0 (188.5–263.5)	234.5 (205.0–268.0)	0.0650
PAAT/√RR	3.18 (2.69–3.76)	3.28 (2.69–3.82)	3.13 (2.67–3.67)	0.2330
PAAT/RVET	0.38 (0.31–0.44)	0.40 (0.34–0.48)	0.36 (0.29–0.41)	0.0001

Population characteristics are expressed as mean ± standard deviation or percentage; arterial-blood gas data and echocardiographic parameters and variables are expressed as median [interquartile range]

APACHE II Acute Physiology and Chronic Health Disease Classification System II, BSA body surface area, FiO_2_ inspired oxygen fraction, HR heart rate, NIV non-invasive ventilation, PAAT pulmonary artery acceleration time, PaCO_2_ arterial partial pressure of carbon dioxide, PaO_2_ arterial partial pressure of oxygen, PEEP positive end-expiratory pressure, RAP right atrial pressure, RR ECG RR interval, RVET right ventricular ejection time, sPAP systolic pulmonary artery pressure, TAPSE tricuspid annular plane systolic excursion

**Table 2:** Characteristics, respiratory variables and echocardiographic parameters of patients admitted to ICU for acute cardiovascular failure and acute respiratory failure

	Cardiovascular failure	Respiratory failure	*p*-value
Population characteristics			
Number of patients	129 (54.7%)	107 (45.3%)	–
Age (years)	68 ± 16	57 ± 17	< 0.0001
Males, n (%)	85 (65.9%)	65 (60.7%)	0.4097
BSA (m^2^)	1.84 ± 0.20	1.89 ± 0.24	0.1339
APACHE II	13.3 ± 5.8	12.5 ± 3.8	0.2460
Ventilation and gas exchanges			
Mechanical ventilation (included NIV), n	100 (77.5%)	104 (92.5%)	0.0017
PEEP (cmH_2_O)	8.0 (6.0–10.0)	10.0 (8.0–12.0)	0.0001
PaO_2_/FiO_2_ (mmHg)	211.5 (179.0–235.0)	168.0 (115.3–194.8)	< 0.0001
PaCO_2_ (mmHg)	40.0 (36.0–45.0)	49.0 (43.0–57.0)	< 0.0001
Echocardiographic parameters and derived variables			
TAPSE (mm)	12.0 (8.4–18.2)	20.0 (15.2–23.7)	< 0.0001
S’ (cm/s)	7.6 (4.7–11.6)	10.2 (8.2–14.5)	0.001
RAP (mmHg)	10.0 (8.0–10.0)	8.0 (8.0–10.0)	0.4751
sPAP (mmHg)	44.0 (38.3–51.9)	52.9 (43.9–63.1)	< 0.0001
PAAT (ms)	85.0 (71.0–95.0)	92.0 (77.0–103.0)	0.0056
Log_10_PAAT	1.93 (1.85–1.98)	1.96 (1.89–2.01)	0.0057
HR (bpm)	90 (80–102)	76 (61–89)	< 0.0001
RVET (ms)	234.5 (202.5–266.0)	221.0 (189.5–265.8)	0.0552
PAAT/√RR	3.18 (2.79–3.73)	3.20 (2.59–3.77)	0.5399
PAAT/RVET	0.36 (0.29–0.41)	0.41 (0.34–0.50)	0.0001

Population characteristics are expressed as mean ± standard deviation or percentage; arterial-blood gas data and echocardiographic parameters and variables are expressed as median (interquartile range)

*APACHE II* Acute Physiology and Chronic Health Disease Classification System II, *BSA* body surface area, *FiO*_*2*_ inspired oxygen fraction, *HR* heart rate, *NIV* non-invasive ventilation, *PAAT* pulmonary artery acceleration time, *PaCO*_*2*_ arterial partial pressure of carbon dioxide, *PaO*_*2*_ arterial partial pressure of oxygen, *PEEP* positive end-expiratory pressure, *RAP* right atrial pressure, RR ECG RR interval, *RVET* right ventricular ejection time, *sPAP* systolic pulmonary artery pressure, *TAPSE* tricuspid annular plane systolic excursion

For the parameters measured, the operator intra‐observer correlation was, respectively, 0.98 (95% confidence interval of 0.978–0.989) and the inter‐observer variability was 0.94 (95% confidence interval of 0.92–0.95).

### Correlation of PAAT with sPAP and TAPSE

#### Overall population

**Fig. 2:**
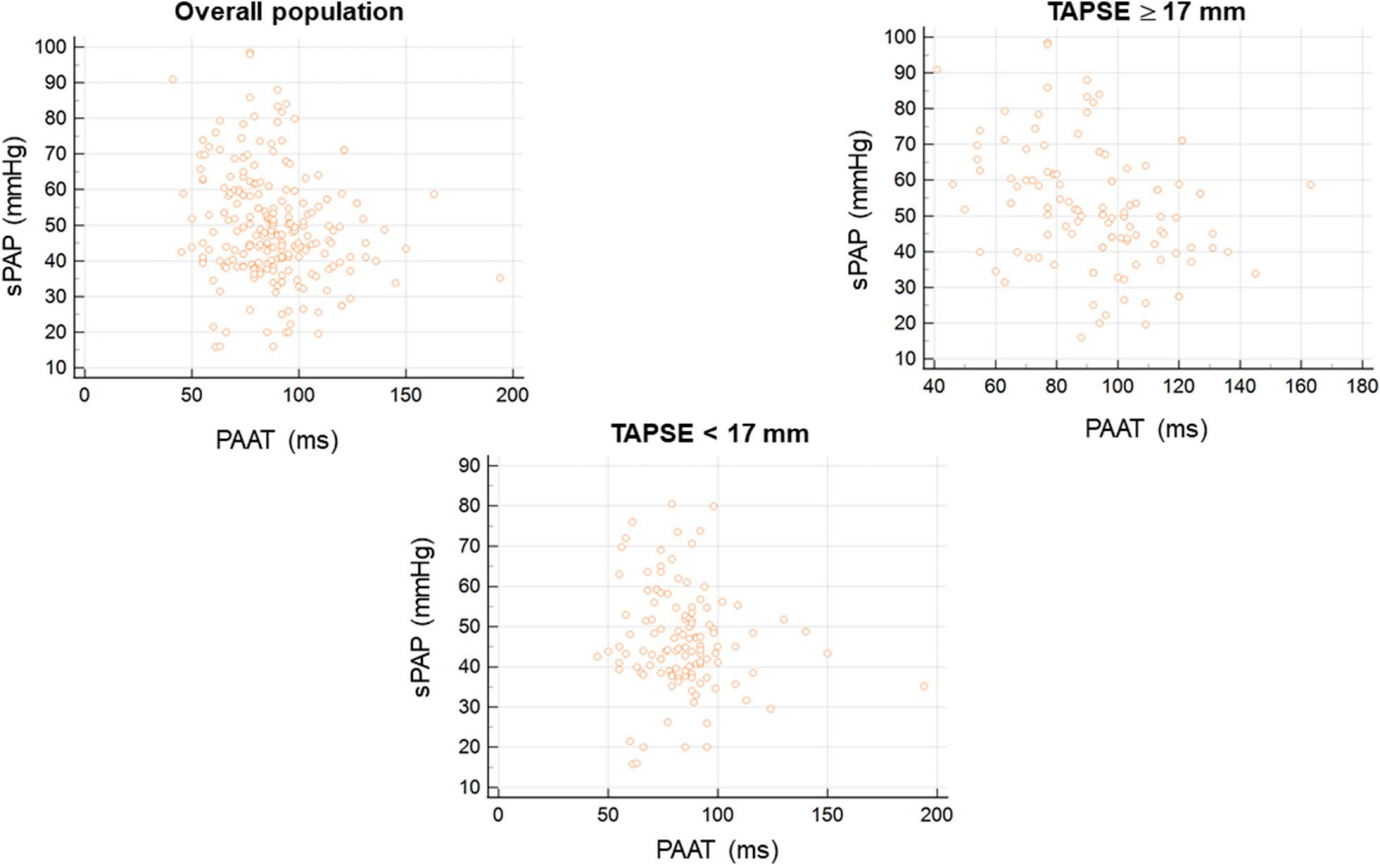
Scatter plot diagrams showing relationship between TR-derived sPAP and PAAT in overall population (left upper panel), preserved RV systolic function (right upper panel) and RV systolic dysfunction (lower panel)

A weak inverse correlation between PAAT and sPAP (ρ 0.189, *p* 0.0035) was observed in overall population (Fig. 2 left upper panel). Adjusting PAAT for its logarithmic correction (log_10_PAAT) and HR did not increase the strength of this relation (respectively, ρ 0.190, *p* 0.0034; ρ 0.237, *p* 0.0002); PAAT adjusted for RVET did not correlate with sPAP (*p* 0.8110).

PAAT had also a weak-positive correlation with TAPSE (ρ 0.197, *p* 0.0024). Adjustment of PAAT for log_10_PAAT, HR and RVET did not yield significative increase in the strength of relationship with TAPSE (respectively, ρ 0.197, *p* 0.0023; *p* 0.6893; ρ 0.264, *p* < 0.0001).

The logistic regression adjusted for covariates including PEEP and reason for admission did not show any significant correlation.

#### Right ventricular systolic function

In patients with preserved RV systolic function, defined as TAPSE ≥ 17 mm and S’ ≥ 9.5 cm/sec, a weak inverse correlation between PAAT and sPAP was found (ρ 0.361, *p* 0.0001) (Fig. 2 right upper panel). This was confirmed either adjusting PAAT for log_10_ (ρ 0.364, *p* 0.0001) and HR (ρ 0.343, *p* 0.0002); no statistically significant correlation was found between PAAT adjusted for RVET and sPAP (*p* 0.1164). We did not find any statistically significant correlation between PAAT and TAPSE (*p* 0.2594), even after adjustment of PAAT for log_10_ (*p* 0.2876), HR (*p* 0.5873) and RVET (*p* 0.5565). The same applied for correlation between S’ and PAAT (*p* 0.134).

In the cohort of patients with impaired RV systolic function, no statistically significant correlation between PAAT and sPAP was demonstrated (p 0.2737) (Fig. 2 lower panel). Logarithmic, HR and RVET corrections of PAAT did not yield statistically significance to the relationship (respectively, *p* 0.2651, *p* 0.0521 and *p* 0.7691). Additionally, PAAT did not correlate with TAPSE (p 0.4515) neither with S’ (p > 0.5). Only adjustment of PAAT for HR yielded a weak inverse correlation with TAPSE (ρ 0.233, *p* 0.0135).

The strength of abovementioned correlations was not influenced by the addition of RAP to TRV_max_ pressure gradient (*p* > 0.5 for all).

#### Role of arterial partial pressure of carbon dioxide

In overall population, a weak-positive correlation between sPAP and PaCO_2_ was observed (ρ 0.268, p < 0.0001), PAAT had a weak-negative correlation with PaCO_2_ (ρ 0.226, *p* 0.0005), and a weak-positive correlation between TAPSE and PaCO_2_ was found (ρ 0.146, *p* 0.0258).

Table 3 shows the correlations, and relative strength, between echocardiographic parameters and PaCO_2_ in patients with preserved and impaired RV systolic function.

**Table 3:** Correlations between echocardiographic parameters and arterial partial pressure of carbon dioxide (PaCO_2_) in patients with preserved and depressed RV systolic function

	TAPSE ≥ 17 mm		TAPSE < 17 mm	
	Correlation coefficient (ρ)	*p*-value	Correlation coefficient (ρ)	*p*-value
sPAP vs. PaCO_2_	0.322	0.0005	0.191	0.0355
PAAT vs. PaCO_2_	−0.385	< 0.0001	–	0.2333
TAPSE vs. PaCO_2_	–	0.9726	0.187	0.0401

*PAAT* pulmonary artery acceleration time, *PaCO*_*2*_ arterial partial pressure of carbon dioxide, *sPAP* systolic pulmonary artery pressure, *TAPSE* tricuspid annular plane systolic excursion

## Discussion

The results of our study demonstrate that PAAT is not a reliable parameter to estimate sPAP in patients admitted to cardiothoracic ICU with and without RV systolic dysfunction.

According to the current guidelines, TVR_max_ is considered the non-invasive parameter of choice for establishing the suspicion of PH. However, TR-methods of estimation of pulmonary artery pressure are burdened with some limitations: (1) difficult measurement of TVR_max_ in trivial or mild TR; (2) sub-optimal Doppler signal alignment with TR jet; (3) underestimation of RA–RV gradient in severe TR, due to the early equalization of chamber pressures [9]; (4) the interrelation between TR peak velocity and RV systolic function; and (5) the inter-observer variability in TR jet quantification [4]*.*

Given the non-negligible pitfalls of TR-derived methods, alternative TR-independent methods, have been proposed for the evaluation of pulmonary artery pressure. It has previously been shown that PAAT should be possible to measure in 99% of patients out of which 25% has no measurable TR and thus provide a way of estimating the pulmonary pressure non-invasively [8]. Different studies, including a recent meta-analysis have demonstrated a reasonable accuracy of PAAT in correctly estimating sPAP and mPAP [3, 16–18].

The evaluation and treatment of RV dysfunction is particularly challenging in critically ill and the coexistence of RV failure and PH is burdened by an increased mortality [19, 20]. While a recent study has assessed the diagnostic accuracy of echocardiography in ventilated patients, no studies were reported of PAAT in patients admitted in ICU with RV dysfunction [21].

Normal PAAT interval values in adults range from 136 to 153 ms [22]. PAAT may be shortened in PH because of a number of reasons: enhanced early pulmonary ejection, increased pulmonary vascular resistance and loss of lung compliance leading to a rapid increase and reduction of flow velocity [15, 23]. PAAT, in fact, represents pulmonary flow acceleration, which increases as the vascular resistance is augmented, based on the Newton law of motion.

Our results are partially in contradiction with the previous literature, as also patients without RV dysfunction did not prove strong relation between Doppler-derived PAAT and TR-estimated sPAP. One of the potentially explaining difference is the admission underlying pathology. Respiratory pathologies may highly influence the interaction between RV and pulmonary circulation system both in settings of normal and impaired RV systolic function [24]. An elegant study on cyclic changes in RV impedance during mechanical ventilation had shown the strict dependence of RV cardiac output and pulmonary artery flow velocity on the ventricular afterload. Noteworthy, the PAAT of those patients were close to normal values during positive-pressure ventilation (104 ms) [25], while our population exhibited lower values.

Arterial partial pressure of carbon dioxide (PaCO_2_) has a well-defined role in determining pulmonary vascular resistances [26, 27], having vasoconstrictive effect on pulmonary circulation. In our study population, we found a positive correlation between TR-derived sPAP and values of PaCO_2_. However, no correlation between PAAT and PaCO_2_ was found in patients with impaired RV systolic function suggesting further caution in the use of PAAT to estimate pulmonary artery pressure.

## Limitations

The present study has a number of limitations: (1) the limited sample size; (2) the heterogeneous population; and (3) sPAP has been assessed only with echocardiography and no validation with pulmonary artery catheter was performed (because of the nature of the observational study and pulmonary artery catheter is not part of the clinical routine armamentarium).

Although echocardiography presents well-known limitations, which are listed in the manuscript, it is considered a reliable diagnostic tool in PH. Additionally, the same methodology has been used in other studies already published and included in the referenced meta-analyses [8, 16].

## Conclusions

sPAP evaluation may be extremely useful in patients with acute respiratory failure, although its estimation based on TR jet may be unfeasible. PAAT measurement to derive sPAP is not reliable in cardiothoracic critically ill patients, particularly in the coexistence of RV systolic impairment. Non-invasive echocardiographic estimation of pulmonary artery pressure in suspected and proven PH remains a challenge, especially in ICU patients. In this specific clinical setting, echocardiographic parameters validated in outpatient population should be adopted with caution.

## Data Availability

The datasets used and analysed during the current study are available from the corresponding author on reasonable request.

## Abbreviations

APACHE II: Acute Physiology and Chronic Health Disease Classification System II
BSA: Body surface area
FiO_2_: Inspired oxygen fraction
HR: Heart rate
ICC: Intra-class correlation coefficient
ICU: Intensive care unit
IVC: Inferior vena cava
NIV: Non-invasive ventilation
mPAP: Mean pulmonary artery pressure
PAAT: Pulmonary artery acceleration time
PaCO_2_: Arterial partial pressure of carbon dioxide
PaO_2_: Arterial partial pressure of oxygen
PEEP: Positive end-expiratory pressure
PH: Pulmonary hypertension
RAP: Right atrial pressure
RR: ECG RR interval
RV: Right ventricle
RVET: Right ventricular ejection time
sPAP: Systolic pulmonary artery pressure
TAPSE: Tricuspid annular plane systolic excursion
TR: Tricuspid regurgitation
TRV_max_: Tricuspid regurgitation velocity peak
